# Inflammatory indexes as predictors of prognosis and bevacizumab efficacy in patients with metastatic colorectal cancer

**DOI:** 10.18632/oncotarget.8901

**Published:** 2016-04-21

**Authors:** Alessandro Passardi, Emanuela Scarpi, Luigi Cavanna, Monia Dall'Agata, Davide Tassinari, Silvana Leo, Ilaria Bernardini, Fabio Gelsomino, Stefano Tamberi, Alba A. Brandes, Elena Tenti, Roberto Vespignani, Giovanni L. Frassineti, Dino Amadori, Ugo De Giorgi

**Affiliations:** ^1^ Department of Medical Oncology, Istituto Scientifico Romagnolo per lo Studio e la Cura dei Tumori (IRST) IRCCS, Meldola, Italy; ^2^ Unit of Biostatistics and Clinical Trials, IRST IRCCS, Meldola, Italy; ^3^ Medical Oncology Unit, Guglielmo da Saliceto Hospital, Piacenza, Italy; ^4^ Department of Oncology, Infermi Hospital, Rimini, Italy; ^5^ Medical Oncology Unit, Vito Fazzi Hospital, Lecce, Italy; ^6^ Medical Oncology Unit, Ramazzini Hospital, Carpi, Italy; ^7^ Oncology Unit, University Hospital Modena, Modena, Italy; ^8^ Oncology Unit, Degli Infermi Hospital, Faenza, Italy; ^9^ Department of Medical Oncology, Azienda USL, Bellaria Hospital - IRCCS Institute of Neurological Sciences, Bologna, Italy; ^10^ Oncology Pharmacy Laboratory, IRST IRCCS, Meldola, Italy; ^11^ IT Service, IRST IRCCS, Meldola, Italy

**Keywords:** metastatic colorectal cancer, bevacizumab, SII NLR PLR, prognosis

## Abstract

**Background:**

To investigate the role of pre-treatment inflammatory indexes (II) as predictors of prognosis and treatment efficacy in patients with metastatic colorectal cancer mCRC randomized onto the prospective multicenter randomized ITACa (Italian Trial in Advanced Colorectal Cancer) trial to receive first-line chemotherapy (CT) with or without bevacizumab (Bev).

**Results:**

In the overall population, PFS and OS were higher in patients with low SII (*p* = .015 and .002, respectively), low NLR (*p* = .0001 and <.0001, respectively) and low PLR (*p* = .004 and .008, respectively). Patients with low NLR in the CT plus Bev arm had a higher PFS than those treated with CT alone (HR = 0.69, *p* = .021).

**Patients and Methods:**

Two hundred and eighty-nine patients were considered for this study, 141 receiving CT plus Bev and 148 receiving CT alone. The pre-treatment systemic immune-inflammation index (SII), neutrophil-to-lymphocyte ratio (NLR) and platelet-lymphocyte ratio (PLR) were evaluated to identify a potential correlation with progression-free (PFS) and overall survival (OS) in both the overall population and the 2 treatment arms.

**Conclusion:**

Our results indicate that II, in particular NLR, are good prognostic and predictive markers for mCRC patients who are candidates for CT plus Bev.

## INTRODUCTION

Bevacizumab (Bev) is a humanized monoclonal antibody with antiangiogenic activity that binds to the vascular endothelial growth factor (VEGF), leading to the inhibition of the circulating ligand and to the prevention of receptor activation [1]. The use of Bev combined with fluoropyrimidine-based chemotherapy (CT) is considered standard first-and second-line treatment for patients with metastatic colorectal cancer (mCRC).

Validated predictors of sensitivity or resistance to Bev are still not available, notwithstanding several studies have investigated this issue in recent years. The majority of these studies focused on the VEGF pathway, including tumor VEGF expression, whereas less attention was paid to the tumor microenvironment and inflammatory response [2].

It has increasingly been recognized that tumor infiltrating inflammatory cells are responsible for producing inflammatory mediators and cytokines that induce angiogenesis, tumor growth, invasion and metastasis [3’^5^]. Accordingly, serum white blood cells, neutrophils, lymphocytes, platelets and acute-phase proteins, such as C-reactive protein and albumin, have been evaluated in different malignancies and found to predict for prognosis and response to treatment [6’^9^]. Moreover, inflammatory indexes (II) obtained with different combinations of these factors, such as neutrophil-to-lymphocyte ratio (NLR) and platelet-lymphocyte ratio (PLR), have been reported to be useful prognostic factors in various malignant solid tumors, including CRC [10’^18^]. The systemic immune-inflammation index (SII) was recently investigated as a prognostic marker in several malignancies including renal cell carcinoma, small cell lung cancer and hepatocellular carcinoma [19’^22^].

In light of the close relationship that has emerged between inflammation and angiogenesis, considerable interest has been aroused in the role of II as predictors of the efficacy of Bev [23]. If validated, these parameters could represent a reproducible, inexpensive and easy method to select candidates for treatment with Bev.

We investigated the prognostic and predictive role of baseline II (SII, NLR and PLR) in mCRC patients treated with first-line CT with or without Bev in the phase III prospective multicenter randomized ITACa (Italian Trial in Advanced Colorectal Cancer) trial (EudraCT no. 2007-004539-44 and on ClinicalTrials.gov (NCT01878422) [24].

## RESULTS

### Patient population

Information on pre-treatment II levels was available for 289 of the 370 patients from the ITACa intention-to-treat population; 145 and 144 had low and high SII values, 168 and 121 had low and high NLR values, and 144 and 145 had low and high PLR values, respectively. Baseline characteristics of patients are shown in Table 1. Patients, divided into groups on the basis of marker cut-offs, were all comparable for age, gender, tumor localization, CT regimen, KRAS status and treatment arm. A higher proportion of patients with high II had a performance status (PS) of 1-2, the high SII and PLR groups included more stage IV tumors at diagnosis, and the high PLR groups had higher grade-tumors.

**Table 1:** Baseline patient characteristics (*n* = 289)

Patient characteristics	NLR		*p*	PLR		*p*	SII		*p*
	<3	≥3		<169	≥169		<730	≥730	
	*n* (%)	*n* (%)		*n* (%)	*n* (%)		*n* (%)	*n* (%)	
**Median age, years (range)**	65 (33-83)	66 (34-81)	.662	66 (33-83)	65 (34-81)	.777	66 (33-83)	65 (34-81)	.351
**Gender**									
Male	103 (61.3)	71 (58.7)		84 (62.2)	90 (58.4)		85 (58.6)	89 (61.8)	
Female	65 (38.7)	50 (41.3)	.653	51 (37.8)	64 (41.6)	.593	60 (41.4)	55 (38.2)	.665
**Performance Status ECOG**									
0	148 (88.1)	82 (67.8)		118 (87.4)	112 (72.7)		127 (87.6)	103 (71.5)	
1-2	20 (11.9)	39 (32.2)	< .0001	17 (12.6)	42 (27.3)	.002	18 (12.4)	41 (28.5)	.0007
**Tumor localisation**									
Rectum	41 (24.4)	36 (29.7)		33 (24.4)	44 (28.6)		39 (26.9)	38 (26.4)	
Colon	127 (75.6)	85 (70.3)	.379	102 (75.6)	110 (71.4)	.429	106 (73.1)	106 (73.6)	.922
**Stage at diagnosis**									
I-III	44 (27.8)	25 (21.2)		40 (31.0)	29 (19.7)		43 (31.6)	26 (18.6)	
IV	114 (72.2)	93 (78.8)	.261	89 (69.0)	118 (80.3)	.031	93 (68.4)	114 (81.4)	.012
**Grade**									
1	9 (6.7)	4 (4.4)		9 (7.9)	4 (3.6)		7 (5.7)	6 (5.8)	
2	92 (68.1)	51 (56.0)		78 (68.4)	65 (58.0)		82 (66.7)	61 (59.2)	
3	34 (25.2)	36 (39.6)	0.068	27 (23.7)	43 (38.4)	0.034	34 (27.6)	36 (34.9)	0.482
**CT regimen**									
FOLFOX4	107 (63.7)	72 (59.5)		77 (57.0)	102 (66.2)		92 (63.4)	87 (60.4)	
FOLFIRI	61 (36.3)	49 (40.5)	.548	58 (43.0)	52 (33.8)	.109	53 (36.6)	57 (39.6)	.596
***KRAS***status**^a^**									
Wild type	96 (61.2)	56 (56.0)		72 (59.0)	80 (59.3)		76 (57.1)	76 (61.3)	
Mutated	61 (38.8)	44 (44.0)	.491	50 (41.0)	55 (40.7)	.968	57 (42.9)	48 (38.7)	.500
**ITACa treatment**									
CT+B	86 (51.2)	55 (45.5)		67 (49.6)	74 (48.1)		75 (51.7)	66 (45.8)	
CT	82 (48.8)	66 (54.5)	.399	68 (50.4)	80 (51.9)	.789	70 (48.3)	78 (54.2)	.317

a Mandatory as consequence of amendment n. 1 of 3 May, 2009. Abbreviations: NRL, neutrophil-to-lymphocyte ratio; PLR, platelet-lymphocyte ratio; SII, systemic immune-inflammation index; ECOG, Eastern Cooperative Oncology Group; ITACa, Italian Trial in Advanced Colorectal Cancer; CT, chemotherapy; B, bevacizumab; n, number.

### Prognostic value of patient characteristics and II

Among patient characteristics, univariate analysis showed that PS was the only variable with a significant impact on survival. Patients with PS = 0 had higher median PFS (9.7 *vs.* 6.8 months; HR = 1.60, 95% CI 1.20-2.15; *p* = .001) and OS (24.8 *vs.* 13.7 months; HR = 2.60, 95% CI 1.90-3.56; *p* < .0001) than those with PS =1-2. No other characteristics correlated with survival (Supplementary Table S1, available online only). Patients with high NLR had a lower median PFS (7.8 *vs.* 10.2 months, *p* = .0001) and lower median OS (16.8 *vs.* 25.2 months, *p* < .0001) than those with low NLR. Patients with high PLR had a lower median PFS (8.3 *vs.* 10.2 months, *p* = .004) and lower median OS (19.0 *vs.* 25.2 months, *p* = .008) than those with high PLR. Patients with high SII levels had a lower median PFS (8.3 *vs.* 10.1 months, *p* < .015) and lower median OS (19.0 *vs.* 25.4 months, *p* = .002) than those with low SII (Table 2).

**Table 2:** Prognostic value of II in the overall population

	No. patients	PFS			HR (95%CI)^a^	*p*	OS			HR (95% CI)^a^	*p*
		No. events	Median PFS (months) (95% CI)	*p*			No. events	Median OS (months) (95% CI)	*p*		
Overall	289	270	9.1 (8.4-9.8)	-	-	-	228	21.3 (19.7-24.5)	-	-	-
NLR <3	168	155	10.2 (9.1-11.3)		1.00		127	25.4 (21.8-31.6)		1.00	
≥3	121	115	7.8 (6.3-8.9)	0.0001	1.58 (1.21-2.07)	0.0009	101	16.8 (13.7-19.9)	<0 .0001	1.68 (1.25-2.27)	0.0006
PLR <169	144	132	10.2 (9.1-11.3)		1.00		111	25.2 (21.3-29.2)		1.00	
≥169	145	138	8.3 (6.9-9.0)	0.004	1.42 (1.09-1.85)	0.008	117	19.0 (16.2-21.4)	0.008	1.50 (1.13-2.00)	0.006
SII <730	145	134	10.1 (9.0-10.9)		1.00		110	25.4 (21.6-29.9)		1.00	
≥730	144	136	8.3 (6.9-9.1)	0.015	1.16 (0.89-1.49)	0.265	118	19.0 (16.2-20.9)	0.002	1.37 (1.03-1.82)	0.030

a adjusted by ITACa treatment, center, CT regimen, KRAS status and baseline characteristics.

Abbreviations: NRL, neutrophil-to-lymphocyte ratio; PLR, platelet-lymphocyte ratio; SII, systemic immune-inflammation index; PFS, progression-free survival; OS, overall survival; HR, hazard ratio; CI, confidence interval.

In multivariable analysis, a backward elimination approach confirmed NLR, tumor localization and PS as independent predictors of PFS (*p* = .001, .064 and .010, respectively) and OS (*p* < .0001, .006 and < .0001, respectively) (Table 3).

**Table 3:** Multivariable analysis of PFS and OS

	PFS		OS	
	HR (95%CI)	p	HR (95% CI)	p
NLR (≥3 *vs.* <3)	1.52 (1.07-2.17)	0.020	1.78 (1.17-2.70)	0.007
PLR (≥169 *vs.* <169)	1.38 (0.99-1.91)	0.051	1.27 (0.89-1.80)	0.186
SII (≥730 *vs.* <730)	0.79 (0.53-1.16)	0.226	0.84 (0.53-1.31)	0.433
Gender (male *vs.* female)	0.97 (0.76-1.25)	0.827	1.04 (0.79-1.37)	0.767
ECOG PS (1-2 *vs.* 0)	1.47 (1.09-1.99)	0.012	2.52 (1.82-3.48)	<0.0001
Tumor localization (colon *vs.* rectum)	1.34 (1.01-1.77)	0.042	1.58 (1.16-2.16)	0.004
CT regimen (FOLFIRI *vs.* FOLFOX4)	1.26 (0.98-1.63)	0.076	1.23 (0.94-1.63)	0.135
KRAS status (mutated *vs.* wild type)	0.98 (0.76-1.26)	0.891	1.07 (0.81-1.41)	0.635
ITACa treatment (CT+B *vs.* CT)	0.84 (0.66-1.07)	0.162	1.28 (0.98-1.67)	0.070
*After backward procedure:*				
NLR (≥3 *vs.* <3)	1.51 (1.18-1.95)	0.001	1.76 (1.33-2.32)	<0.0001
Gender (male *vs.* female)	0.95 (0.74-1.22)	0.677	1.02 (0.77-1.34)	0.906
ECOG PS (1-2 *vs.* 0)	1.48 (1.10-2.00)	0.010	2.51 (1.82-3.47)	<0.0001
Tumor localization (colon *vs.* rectum)	1.30 (0.98-1.71)	0.064	1.54 (1.13-2.09)	0.006
CT regimen (FOLFIRI *vs.* FOLFOX4)	1.19 (0.93-1.53)	.167	1.18 (0.90-1.55)	.217
KRAS status (mutated *vs.* wild type)	1.00 (0.78-1.28)	.995	1.08 (0.82-1.42)	.592
ITACa treatment (CT+B *vs.* CT)	0.85 (0.66-1.09)	.193	1.29 (0.98-1.68)	.064

Abbreviations: NRL, neutrophil-to-lymphocyte ratio; PLR, platelet-lymphocyte ratio; SII, systemic immune-inflammation index;

PFS, progression-free survival; OS, overall survival; HR, hazard ratio; CI, confidence interval; ECOG, Eastern Cooperative Oncology Group;

PS, performance status; ITACa, Italian Trial in Advanced Colorectal Cancer; CT, chemotherapy; B, bevacizumab

### Predictive value of the II

Results of the impact of treatment (CT plus Bev and CT alone) on PFS and OS according to the analyzed II, together with 95% CI and HR data, are summarized in Table 4.

**Table 4:** Predictive value of II in the CT plus Bev and CT-only treatment arms

	No. patients	PFS			HR (95%CI)^a^	*p*	OS			HR (95% CI)^a^	*p*
		No. events	Median PFS (months) (95% CI)	*p*			No. events	Median OS (months) (95% CI)	*p*		
NLR											
*CT+B*											
NLR <3	86	77	12.4 (10.3-14.0)		1.00		65	30.4 (22.6-36.1)		1.00	
≥3	55	54	6.9 (4.7-9.0)	< 0.0001	2.27 (1.53-3.37)	< 0.0001	48	12.7 (7.9-15.3)	< 0.0001	2.48 (1.61-3.83)	< 0.0001
*CT*											
NLR <3	82	78	8.9 (7.2-9.8)		1.00		62	24.3 (20.2-28.0)		1.00	
≥3	66	61	8.0 (6.2-9.1)	0.315	1.12 (0.77-1.62)	0.556	53	21.3 (16.8-24.5)	0.143	1.19 (0.78-1.81)	0.415
PLR											
*CT+B*											
PLR <169	72	64	11.4 (9.8-13.4)		1.00		56	27.0 (20.6-34.5)		1.00	
≥169	69	67	8.8 (6.4-9.9)	0.006	1.62 (1.09-2.40)	0.017	57	15.9 (12.9-20.9)	0.061	1.67 (1.09-2.56)	0.019
*CT*											
PLR <169	72	68	9.3 (8.3-10.3)		1.00		55	24.8 (20.3-29.2)		1.00	
≥169	76	71	7.3 (5.5-8.9)	0.158	1.26 (0.88-1.80)	0.214	60	20.4 (16.8-24.5)	0.106	1.39 (0.93-2.08)	0.105
SII											
*CT+B*											
SII <730	75	68	11.5 (9.8-13.2)		1.00		58	27.4 (21.8-34.5)		1.00	
≥730	66	63	8.6 (6.4-9.9)	0.014	1.41 (0.97-2.06)	0.072	55	15.1 (11.6-19.3)	0.002	1.68 (1.10-2.57)	0.016
*CT*											
SII <730	70	66	9.0 (7.0-9.8)		1.00		52	24.8 (20.2-29.9)		1.00	
≥730	78	73	8.1 (6.5-9.1)	0.408	0.99 (0.70-1.42)	0.978	63	20.4 (17.1-24.3)	0.114	1.21 (0.80-1.82)	0.369

a adjusted by ITACa treatment, center, CT regimen, KRAS status and baseline characteristics.

Abbreviations: NRL, neutrophil-to-lymphocyte ratio; PLR, platelet-lymphocyte ratio; SII, systemic immune-inflammation index; PFS, progression-free survival; OS, overall survival; HR, hazard ratio; CI, confidence interval; CT, chemotherapy; B, bevacizumab.

### SII

Median PFS in the CT plus Bev group was 11.5 (95% CI 9.8-13.2) and 8.6 (95% CI 6.4-9.9) months in patients with low and high SII, respectively (*p* = .014), while in the CT-only arm it was 9.0 (95% CI 7.0-9.8) and 8.1 (95% CI 6.5-9.1) months in patients with low and high SII, respectively (*p* = .408). Median OS was significantly associated with SII levels in the CT plus Bev group (27.4 *vs.*15.1 months in low and high SII patients, respectively, *p* = .002), but not in the CT-only arm (24.8 *vs.* 20.4 months, *p* = .114). The interaction test did not reveal a significant correlation between SII levels on the basis of cut-off and treatment for either PFS or OS (*p* = .290 and .279, respectively). In contrast, the evaluation of SII as a continuous variable showed a positive interaction test for both PFS (*p* = .033) and OS (*p* = .043).

### NLR

In the CT plus Bev group, median PFS was 12.4 (95% CI 10.3-14.0) and 6.9 (95% CI 4.7-9.0) months in patients with low and high NLR, respectively (*p* < .0001), and median OS was 30.4 (95% CI 22.6-36.1) and 12.7 (95% CI 7.9-15.3), respectively (*p* < .0001). In the CT-only arm, median PFS was 8.9 (95% CI 7.2-9.8) and 8.0 (95% CI 6.2-9.1) months in patients with low and high NLR, respectively (*p* = .315), and median OS was 24.3 (95% CI 20.2-28.0) and 21.3 (95% CI 16.8-24.5), respectively (*p* = .143).

The interaction test involving NLR levels and the effect of treatment in either group suggested that the correlation between NLR levels and improved outcome was significantly associated with the addition of Bev for both PFS (HR 1.75; 95% CI 1.08-2.84; *p* = .024) and OS (HR = 1.90; 95% CI 1.12-3.22; *p* = .017). This association was confirmed by evaluating the index as a continuous variable (PFS, *p* = .022; OS, *p* = .013).

### PLR

In the CT plus Bev group, median PFS was 11.4 (95% CI 9.8-13.4) and 8.8 (95% CI 6.4-9.9) months in patients with low and high PLR, respectively (*p* = .006), and median OS was 27.0 and 15.9 months (*p* = .061). In the CT-only arm, median PFS was 9.3 (95% CI 8.3-10.3) and 7.3 (95% CI 5.5-8.9) months in patients with low and high PLR, respectively (*p* = .158), and median OS was 24.8 and 20.4 months, respectively (*p* = .106). The interaction tests, which considered the cut-off or the continuous variable, did not show any significant correlation between PLR levels and the effect of Bev on outcome (data not shown).

### Efficacy of Bev as a function of II

**Figure 1:**
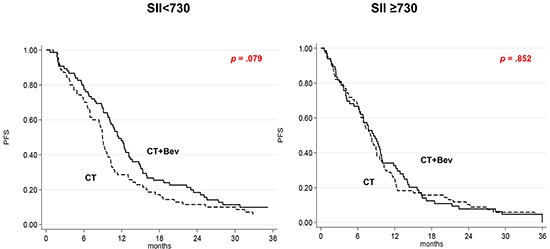
Kaplan-Meier curves of progression-free survival according to treatment as a function of SII

**Figure 2:**
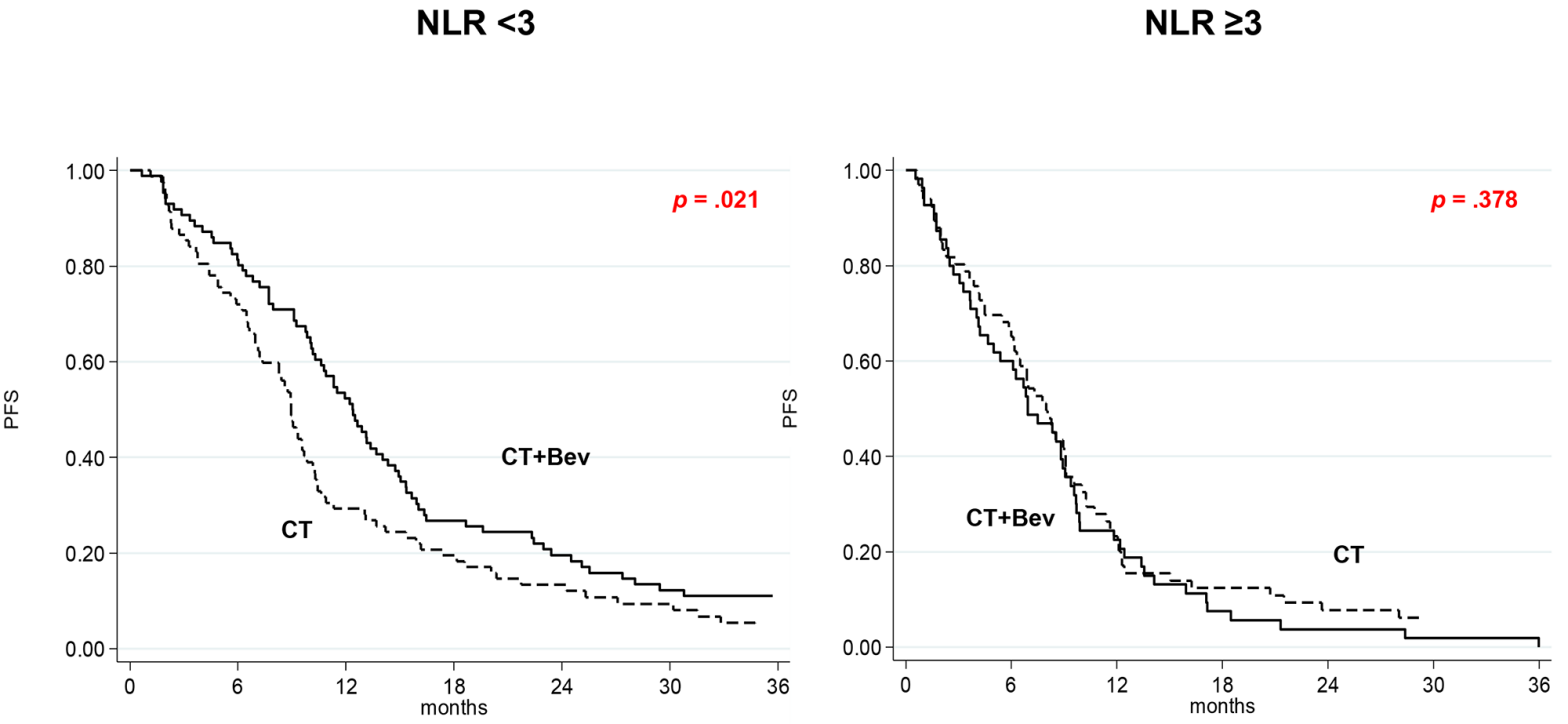
Kaplan-Meier curves of progression-free survival according to treatment as a function of NLR

The effect of adding Bev to CT as a function of II was also investigated. Among patients with low SII, a higher, albeit non significant, PFS was observed in those treated with CT plus Bev than in those receiving CT alone (HR = 0.74, 95% CI 0.52-1.04; *p* = .079). In high SII patients, PFS did not differ between the 2 treatment arms (HR = 0.97, 95% CI 0.69-1.36; *p* = .852) (Figure 1). Treatment with Bev did not lead to improved OS in patients with either high or low SII values (Supplementary Figure S1, available online only). Patients with low NLR in the CT plus Bev arm had a higher PFS than those treated with CT alone (HR 0.69, 95% CI 0.50-0.94, *p* = .021) (Figure 2), while Bev-treated patients with high NLR had a poorer OS than those receiving CT alone (HR = 1.69, 95% CI 1.14-2.51, *p* = .009) (Supplementary Figure S2, available online only).

**Figure 3:**
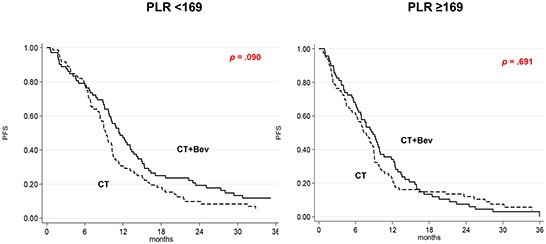
Kaplan-Meier curves of progression-free survival according to treatment as a function of PLR

Patients with low PLR baseline values in the CT plus Bev arm had a trend towards higher PFS than those in the CT-only arm (HR = 0.74, 95% CI 0.53-1.05; *p* = .090), while those with high PLR showed a similar PFS in both treatment arms (HR = 0.93, 95% CI 0.67-1.31; *p* = .691) (Figure 3). OS was not affected by the addition of Bev in patients with either high or low PLR values (Supplementary Figure S3, available online only).

## DISCUSSION

Inflammation produced by the secretion of cytokines and chemokines promotes tumor growth, angiogenesis and metastasis [3]. Several studies have shown that platelets induce circulating tumor cell epithelial-mesenchymal transition and promote extravasation to metastatic sites [25, 26]. Neutrophils promote adhesion and seeding of distant organ sites through the secretion of circulating growth factors such as VEGF and proteases [27, 28]. Lymphocytes play a crucial role in tumor defense by inducing cytotoxic cell death and inhibiting tumor cell proliferation and migration, thereby dictating the host's immune response to malignancy [29]. Thus, inflammation induces changes in the cancer microenvironment that favor cancer progression.

In light of this, several II have been investigated as possible predictors of prognosis and response to treatment in different tumor types. Among these, NLR and PLR represent the most common indices [10’^18^], while the SII was only recently introduced [19’^22^]. Compared with other potential markers, the measurement of these parameters has the advantage of being inexpensive and reproducible.

The aim of our study was to evaluate the potential usefulness of these II to estimate prognosis and to predict the efficacy of treatment with Bev. Univariate analysis in the overall population showed that, in addition to performance status, SII, NLR, and PLR were significantly associated with PFS and OS, whereas in multivariate analysis, only ECOG PS and NLR remained markers of PFS and OS.

The impact of adding Bev to CT differed on the basis of the II and the incorporation (PLR and SII) or not (NLR) of the platelet count. In the present study, NLR appeared to be the most powerful indicator of prognosis. Patients with high NLR treated with Bev had a poorer OS than those treated with CT alone, whereas the addition of Bev did not lead to any significant difference in OS in the SII or PLR groups. Moreover, patients with low NLR in the CT plus Bev arm had a higher PFS than those treated with CT alone.

In addition to promoting tumor angiogenesis, there is evidence that VEGF favors tumor immune evasion and immune response suppression through different mechanisms mainly regulated by myeloid-derived suppressor cells (MDSCs) [30]. Bev, as an anti-VEGF agent, may thus induce immune response through several mechanisms including increased trafficking of T cells into tumors, reduction of suppressive cytokines and tumor-infiltrating T regulatory cells and MDSCs, increased CD8+ and CD4+ central memory T cells, and reduced frequency of MDSCs [31’^34^]. Whilst the inhibition of VEGF signaling appears to enhance CT efficacy in front-line treatment of patients with low II, high NLR is associated with a poor prognosis and may be correlated with a detrimental immunological effect of Bev. Overall, the role of VEGF in the immune response and its critical role in CRC pathogenesis may represent a rationale to test whether the inhibition of the PD-L1/PD-1 pathway by immune checkpoint inhibitors in combination with anti-VEGF therapies enhances clinical response in mCRC patients. The combined use of II and histological biomarkers (*e.g.* PDL1 expression) could help to select suitable candidates for these treatments.

In conclusion, II are powerful prognostic and predictive indicators of poor outcome in mCRC patients treated with CT +/− Bev. The addition of Bev to CT only appears to improve clinical outcome in those with favorable II values. Validation in a larger prospective data set is warranted.

## MATERIALS AND METHODS

### Patient population and treatment regimens

Two hundred and eighty-nine patients enrolled onto the first-line ITACa trial were considered. The study design and key eligibility and exclusion criteria have previously been described in detail [24]. Patients were recruited from 14^th^ November 2007 to 6^th^ March 2012 and followed up until 31^st^ December 2013. After randomization, 176 patients underwent CT (either FOLFIRI or FOLFOX4) plus Bev, while 194 patients received CT alone. Patients were treated until disease progression or unacceptable toxicity occurred. All patients provided written informed consent. The study was carried out in accordance with the Declaration of Helsinki under good clinical practice conditions and after ethics committee approval of all participating centers. Tumor response was radiologically evaluated every 8 weeks according to the Response Evaluation Criteria in Solid Tumors (RECIST) until disease progression or withdrawal. The primary endpoint of the trial was progression-free survival (PFS) and secondary endpoints included overall survival (OS).

Information on neutrophil, lymphocyte and platelet counts from blood tests carried out at baseline (before systemic treatment) was collected. SII was calculated as platelet count × neutrophil count/lymphocyte count, NLR was obtained by dividing the absolute neutrophil count by the absolute lymphocyte count, and PLR was calculated as the ratio of absolute platelet count to absolute lymphocyte count.

### Statistical analysis

The aim of this secondary analysis was to examine the association between baseline II levels and PFS and OS in the overall population, and separately in the 2 treatment arms. The data cut-off for analysis was 31^st^ December 2013 when the median duration of follow-up was 36 months (range 1-65). PFS was defined as the time from random assignment to the first documentation of PD (per investigator assessment), or death from any cause. Patients undergoing curative metastasectomy were censored at the time of surgery. OS was defined as the time interval between random assignment and death or last follow-up visit. PFS and OS were estimated by the Kaplan-Meier method and curves were compared by the log-rank test (at a significance level of 5%). Estimated hazard ratios (HRs) and their two-sided 95% Confidence Intervals (95% CI) were calculated using the Cox proportional-hazard model. HRs adjusted by center and baseline characteristics (gender, age, ECOG performance status, *KRAS* status, tumor localization (rectum/colon) and CT regimen (FOLFOX4/FOLFIRI) were calculated using the Cox proportional hazards model. Covariate selection was based on a list of suspected prognostic factors derived from the ITACa study [24].

The effect of the interaction between II levels and treatment on PFS/OS was evaluated using Cox regression models for the entire population (CT+B and CT-only arms) including II levels, treatment and treatment-by-II levels. X-tile 3.6.1 software (Yale University, New Haven, CT) was used for bioinformatic analysis of baseline data to determine the cutoff value for pre-treatment levels of each II. SII ≥730, NLR ≥3 and PLR ≥169 were considered as elevated levels.

All *p* values were based on two-sided testing and statistical analyses were performed using SAS statistical software version 9.4 (SAS Inc., Cary, NC, USA).

## SUPPLEMENTARY FIGURES AND TABLE




