# Supplementary material for: Inflammatory indexes as predictors of prognosis and bevacizumab efficacy in patients with metastatic colorectal cancer
Source: Oncotarget. 2016 Apr 21;7(22):33210–9. doi: 10.18632/oncotarget.8901 (PMC5078087; doi:10.18632/oncotarget.8901)
Supplement: Supplementary file 1 [file oncotarget-07-33210-s001.pdf]

SUPPLEMENTARY FIGURES AND TABLE

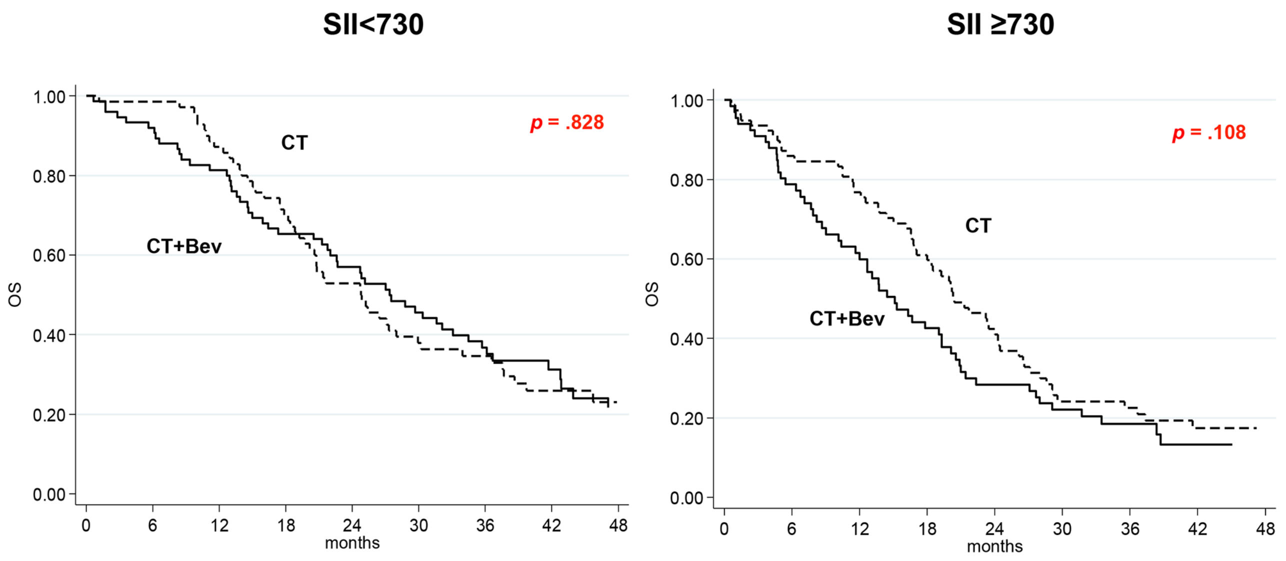

Supplementary Figure S1: Kaplan-Meier curves of overall survival according to treatment as a function of SII.

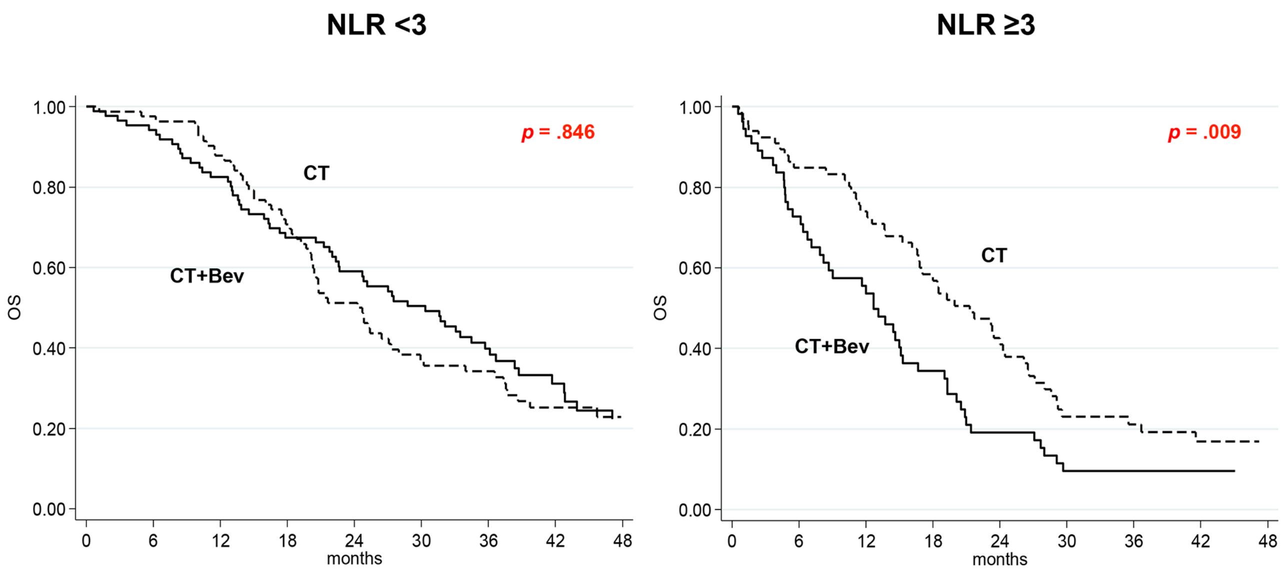

Supplementary Figure S2: Kaplan-Meier curves of overall survival according to treatment as a function of NLR.

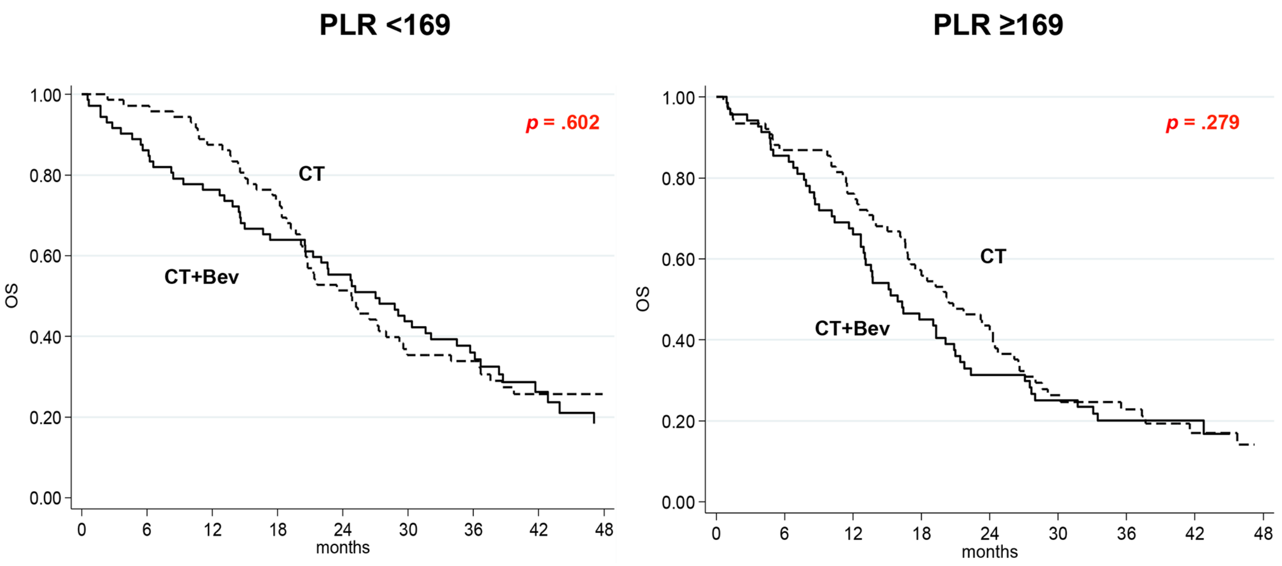

Supplementary Figure S3: Kaplan-Meier curves of overall survival according to treatment as a function of PLR.

Supplementary Table S1: Progression-Free Survival and Overall Survival according to patient characteristics (*n* = 289).

See Supplementary File 1
