# Supplementary material for: Inflammatory indexes as predictors of prognosis and bevacizumab efficacy in patients with metastatic colorectal cancer
Source: Oncotarget. 2016 Apr 21;7(22):33210–9. doi: 10.18632/oncotarget.8901 (PMC5078087; doi:10.18632/oncotarget.8901)
Supplement: Supplementary file 2 [file oncotarget-07-33210-s002.doc]

**Table S1.** Progression-Free Survival and Overall Survival according to patient characteristics (*n* = 289)

|  | ***n* patients** | ***n***  **events** | **Median PFS (months)**  **(95% CI)** | **HR (95% CI)** | ***p*** | ***n* events** | **Median OS (months)**  **(95% CI)** | **HR (95% CI)** | ***p*** |
| --- | --- | --- | --- | --- | --- | --- | --- | --- | --- |
| **Gender** |  |  |  |  |  |  |  |  |  |
| Female | 115 | 107 | 9.1 (7.4-10.1) | 1.00 |  | 91 | 20.6 (16.8-24.9) | 1.00 |  |
| Male | 174 | 163 | 9.1 (8.3-10.2) | 0.93 (0.73-1.19) | .570 | 137 | 22.3 (19.7-25.2) | 0.97 (0.74-1.27) | .833 |
| **Performance Status** (ECOG) |  |  |  |  |  |  |  |  |  |
| 0 | 230 | 212 | 9.7 (8.9-10.4) | 1.00 |  | 173 | 24.8 (21.3-27.5) | 1.00 |  |
| 1-2 | 59 | 58 | 6.8 (3.8-8.9) | 1.60 (1.20-2.15) | .001 | 55 | 13.7 (9.7-16.8) | 2.60 (1.90-3.56) | < .0001 |
| **Tumor localization** |  |  |  |  |  |  |  |  |  |
| Rectum | 77 | 73 | 9.9 (8.5-12.0) | 1.00 |  | 59 | 27.4 (21.7-33.1) | 1.00 |  |
| Colon | 212 | 197 | 8.9 (7.5-9.7) | 1.20 (0.92-1.57) | .182 | 169 | 20.2 (17.5-22.0) | 1.36 (1.01-1.82) | .045 |
| **Stage at diagnosis** |  |  |  |  |  |  |  |  |  |
| I-III | 69 | 65 | 10.2 (9.1-12.0) | 1.00 |  | 52 | 28.0 (21.3-34.5) | 1.00 |  |
| IV | 207 | 193 | 8.9 (8.0-9.6) | 1.12 (0.85-1.49) | .422 | 164 | 20.3 (17.5-23.3) | 1.33 (0.97-1.82) | .075 |
| **Grade** |  |  |  |  |  |  |  |  |  |
| 1 | 13 | 13 | 9.6 (6.5-13.4) | 1.00 |  | 10 | 18.4 (13.3-27.4) | 1.00 |  |
| 2 | 143 | 132 | 9.7 (8.8-10.9) | 0.81 (0.46-1.44) | 0.260 | 111 | 27.0 (21.4-28.8) | 0.76 (0.40-1.46) | 0.219 |
| 3 | 70 | 65 | 8.5 (6.2-9.5) | 1.04 (0.57-1.88) |  | 56 | 19.1 (13.8-24.0) | 1.00 (0.51-1.96) |  |
| **CT regimen** |  |  |  |  |  |  |  |  |  |
| FOLFOX4 | 179 | 164 | 9.7 (8.8-10.4) | 1.00 |  | 134 | 21.3 (18.4-27.1) | 1.00 |  |
| FOLFIRI | 110 | 106 | 8.6 (7.0-9.2) | 1.20 (0.94-1.54) | .137 | 94 | 21.4 (18.5-24.7) | 1.26 (0.97-1.65) | .082 |
| ***KRAS* status** |  |  |  |  |  |  |  |  |  |
| Wild type | 184 | 171 | 8.6 (7.5-9.3) | 1.00 |  | 145 | 20.9 (18.2-24.7) | 1.00 |  |
| Mutated | 105 | 99 | 10.0 (9.0-11.3) | 0.95 (0.74-1.22) | .701 | 83 | 22.3 (19.9-27.0) | 1.00 (0.76-1.31) | .979 |
| **Prior cancer therapy** |  |  |  |  |  |  |  |  |  |
| Surgery: No | 74 | 69 | 7.7 (6.0-9.1) | 1.00 |  | 58 | 16.3 (13.1-21.3) | 1.00 |  |
| Yes | 215 | 201 | 9.5 (8.9-10.3) | 0.79 (0.60-1.04) | .091 | 170 | 23.2 (20.8-27.1) | 0.80 (0.59-1.08) | .147 |
| Radiotherapy: No | 261 | 244 | 9.0 (8.3-9.7) | 1.00 |  | 206 | 20.8 (18.5-23.2) | 1.00 |  |
| Yes | 28 | 26 | 10.1 (6.9-15.9) | 0.86 (0.57-1.29) | .469 | 22 | 29.1 (20.8-42.8) | 0.74 (0.48-1.15) | .180 |
| Adjuvant chemotherapy: No | 248 | 232 | 9.0 (8.3-9.7) | 1.00 |  | 195 | 20.8 (18.8-24.3) | 1.00 |  |
| Yes | 41 | 38 | 9.8 (6.5-12.4) | 0.97 (0.69-1.37) | .856 | 33 | 27.1 (19.3-36.1) | 0.89 (0.62-1.29) | .558 |
| **ITACa treatment** |  |  |  |  |  |  |  |  |  |
| CT | 148 | 139 | 8.6 (7.2-9.1) | 1.00 |  | 115 | 21.7 (19.9-25.2) | 1.00 |  |
| CT+B | 141 | 131 | 9.9 (8.9-11.8) | 0.83 (0.65-1.05) | .118 | 113 | 20.9 (15.9-25.2) | 1.15 (0.88-1.49) | .299 |
